# Bioactive Plant Compounds in Coffee Charcoal (*Coffeae carbo*) Extract Inhibit Cytokine Release from Activated Human THP-1 Macrophages

**DOI:** 10.3390/molecules24234263

**Published:** 2019-11-22

**Authors:** Laura Schiller, Dima Hammoud Mahdi, Steffen Jankuhn, Bartosz Lipowicz, Cica Vissiennon

**Affiliations:** 1 Institute of Medical Physics and Biophysics, Medical Faculty, Leipzig University, 04107 Leipzig, Germany; 2 Repha GmbH Biologische Arzneimittel, 30855 Langenhagen, Germany; 3 Inter-Regional University of Industrial Engineering Biotechnologies and Applied Sciences, IRGIB Africa University, Cotonou 07 BP 231, Benin; 4 Felix Bloch Institute for Solid State Physics, Faculty of Physics and Earth Sciences, Leipzig University, 04103 Leipzig, Germany

**Keywords:** coffee charcoal, *Coffeae carbo*, *Coffea arabica*, pharmacology, inflammation, gastrointestinal disorder, IBD

## Abstract

The herbal preparation coffee charcoal is produced by over-roasting and milling green dried *Coffea arabica* L. seeds, and has a long-standing tradition in the treatment of inflammatory and gastrointestinal disorders. Its therapeutic properties are commonly attributed to adsorptive and astringent effects. This insufficiently explains its mode of action, especially when used in the treatment of inflammatory diseases in lower dosages. Our investigations aimed to identify bioactive secondary plant metabolites affecting cytokine-signaling. Thus, a phytochemical analysis of coffee charcoal extract was conducted using HPLC and LC/MS. Trigonelline, neochlorogenic acid, chlorogenic acid, caffeine, cryptochlorogenic acid, feruloylquinic acid isomers, and a caffeoylquinolacton were identified in the extract. Subsequently, the effects of coffee charcoal extract, chlorogenic acid isomers, their metabolite caffeic acid, caffeine, and trigonelline on cytokine (TNF, IL-6, MCP-1) release from LPS-challenged human THP-1 macrophages were examined to evaluate anti-inflammatory activity. Coffee charcoal showed concentration-dependent mild-to-medium inhibitory effects. The chlorogenic acid isomers and caffeic acid inhibited the TNF release, with cryptochlorogenic acid exerting the most distinct effects, as well as decreasing the release of IL-6 and MCP-1. In addition, scanning electron microscopic images provided an impression of the particle constitution, indicating a larger particle size and less structured surface of coffee charcoal in comparison to activated charcoal. In conclusion, our findings underline that beyond adsorptive effects, coffee charcoal exhibits pharmacological properties, which derive from a spectrum of secondary plant metabolites and support the therapeutic use in inflammatory diseases. Chlorogenic acids, particularly cryptochlorogenic acid, appear as pivotal bioactive compounds.

## 1. Introduction

Coffee charcoal, also referred to as *Coffeae carbo*, is a herbal preparation with a long tradition in the treatment of inflammatory disorders. It was introduced into medical practice in 1937 by August G. Heisler [1]. Coffee charcoal is produced by roasting green dried *Coffea arabica* L. seeds to blackening on the outside and milling to powder. Although the name might suggest it, the resulting product is not a charred, carbonized charcoal, but rather over-roasted coffee, which still contains a considerable amount of secondary plant substances like caffeine, trigonelline, chlorogenic acid, and caffeic acid^2^. Traditional areas of application range from angina, paradontosis, and migraine to wound care, diarrhea, and other functional and inflammatory disorders of the gastrointestinal tract [1,2,3,4,5]. 

Nowadays, coffee charcoal is mainly used in the treatment of diarrhea [6] and as part of a combination product (Myrrhinil-Intest^®^) with myrrh and chamomile flower [7] as a treatment option for various gastrointestinal disorders. Recent observational and clinical studies reinforce the use of the traditional herbal combination in these indications, and particularly as a therapeutic option for inflammatory bowel diseases [8,9,10]. 

The German Commission E monograph on coffee charcoal considers its adsorptive and astringent effects to cause the therapeutic effects at the recommended average daily dose of 9 g [11]. Additionally, and in particular, when coffee charcoal is applied in smaller doses within a herbal combination, an underlying pharmacological activity of the plant compounds contained is proposed to contribute to the therapeutic properties [12,13,14]. 

In vitro experiments showed that coffee charcoal extract caused a downregulation of immune response-associated gene expression in LPS- and IFNγ-challenged human monocyte-derived macrophages. These investigations further revealed a decrease of the pro-inflammatory TNF and CXCL-13 and an increase of anti-inflammatory IL-10 release from activated THP-1 macrophages by coffee charcoal [12]. The underlying mechanisms and active components determining this effect remain to be elucidated. This study aimed to identify pharmacologically active secondary plant metabolites with respect to cytokine-signaling. Thus, a phytochemical characterization of coffee charcoal extract was conducted using HPLC and LC/MS. Afterwards, the anti-inflammatory properties of respective substances were evaluated using LPS-challenged human macrophages. In addition, scanning electron microscopic (SEM) images of coffee charcoal and activated charcoal were produced to compare the microscopic constitution of their particles with regard to surface structure, porosity, and presence of pore ducts. 

## 2. Results

### 2.1. HPLC and LC/MS Characterization of the Extract

**Figure 1:**
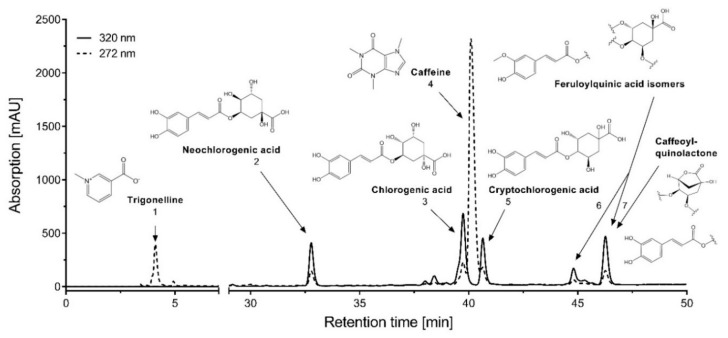
HPLC chromatogram of coffee charcoal extract with chemical structures of identified substances.

Using HPLC and LC/MS analysis of the coffee charcoal extract, seven peaks were assigned to plant compounds, as presented within Figure 1. The chromatographic, UV–Vis spectroscopic and mass spectrometric characteristics are summarized in Table 1. Through comparison with literature data [15,16,17] and subsequent confirmation with reference substances, the peaks 1–5 were assigned to trigonelline, neochlorogenic acid, chlorogenic acid, caffeine, and cryptochlorogenic acid. The assignment of peaks 6 and 7 to feruloylquinic acid isomers, and in the case of peak 7, an additional caffeoylquinolactone was based on corresponding literature data [15,16].

### 2.2. Effects on Cytokine Release from Activated Macrophages

**Figure 2:**
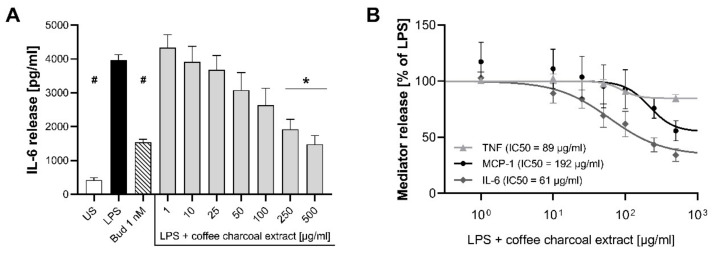
Effects of coffee charcoal extract (1–500 µg/mL) on LPS-induced inflammatory mediator release from THP-1 macrophages. (**A**) Absolute IL-6 release; (**B**) concentration response curve for the inhibition of TNF, IL-6, and MCP-1 release; US = unstimulated control, LPS = LPS-stimulated and untreated control, Bud = LPS stimulation and budesonide 1 nM. Mean ± standard error of the mean (SEM), *n* = 9–11, ^#^ *p* < 0.001, * *p* < 0.01 compared to LPS in an ordinary one-way ANOVA.

LPS-stimulation led to a 6.3-fold higher TNF, 9.3-fold higher IL-6, and 2.2-fold higher MCP-1 release from THP-1 macrophages compared to the unstimulated control. The LPS-induced pro-inflammatory mediator release was inhibited by the positive control budesonide 1 nM (42 ± 3% TNF, 61 ± 2% IL-6, 52 ± 11% MCP-1, *p* < 0.005, *n* = 8–11). Coffee charcoal extract exerted concentration-dependent inhibitory effects on the release of all three mediators with mild (+) to medium (++) maximum inhibition values (*p* < 0.05, *n* = 9–11) of 15 ± 3% for TNF (IC50 = 89 [95% CI 55.14–206.9] µg/mL), 66 ± 6% for IL-6 (IC50 = 61 [95% CI 36.68–98.01] µg/mL), and 44 ± 7% for MCP-1 (IC50 = 192 [95% CI 69.71–1177] µg/mL,) in the highest concentration of 500 µg/mL (Figure 2). 

The inhibitory effects of the pure plant compounds, which were assessed within the same experimental set-up, are summarized and color-coded based on effect size ranking in Table 2.

Cell viability was not altered by LPS stimulation and treatment with test substances compared to the unstimulated control within the MTT assay.

### 2.3. SEM Images of Coffee Charcoal and Activated Charcoal

**Figure 3:**
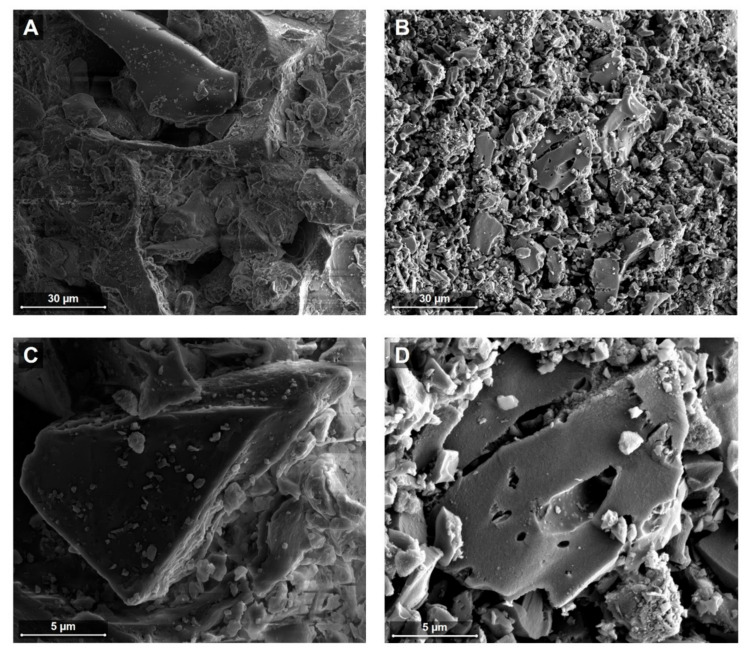
Scanning electron microscopic images of coffee charcoal (**A**,**C**) and activated charcoal (**B**,**D**) obtained with dual beam microscope (DBM) in the low voltage mode at 5 kV with horizontal field widths of 85.3 µm (**A**,**B**) and 21.3 µm (**C**,**D**).

Scanning electron microscopic images of coffee charcoal and activated charcoal were obtained to provide a visualization of the particle constitution. A qualitative comparison indicates that the activated charcoal sample showed a smaller particle size and increased porosity with more distinct pore ducts than coffee charcoal, as visible in the representative images displayed in Figure 3.

## 3. Discussion

The application of coffee charcoal in the treatment of inflammation and gastrointestinal disorders is based on a long-standing tradition. To gain a better understanding about the mechanisms eliciting the therapeutic qualities of coffee charcoal, this study aimed to identify secondary plant metabolites and examine their influence on pro-inflammatory mediator release from activated macrophages.

Using HPLC and LC/MS analysis, trigonelline, caffeine, caffeoylquinic acids, feruloylquinic acids, as well as a caffeoylquinolactone were detected in an aqueous coffee charcoal extract. These findings are in line with an early study on the phytochemical composition of this herbal medicinal product that mentioned trigonelline, caffeine, chlorogenic and caffeic acid as components [3]. The additional substances we identified within the coffee charcoal extract correspond with the spectrum of secondary plant metabolites described for green and roasted coffee [15,18]. This substantiates the fact that the over-roasting process does not lead to complete carbonization, but rather, results in a distinct herbal preparation, with active components of potential pharmacological relevance. Early investigations reported that the adsorptive properties of coffee charcoal are increased throughout the roasting process, but range below the level of activated charcoal, thus the author suggested an underlying pharmacological activity to account for its therapeutic effects [3,4,19]. Scanning electron microscopic images obtained in this study indicate differences in the particle constitution, with a larger particle size and less structured surface of coffee charcoal compared to activated charcoal, which might explain its lower adsorption capacity. 

Mild-to-medium inhibitory effects of coffee charcoal extract on pro-inflammatory TNF, IL-6, and MCP-1 release from LPS-challenged THP-1 macrophages were observed in the second part of the study. This reinforces the suggested anti-inflammatory properties of the herbal product from previous pharmacological studies [12]. To elucidate which secondary plant metabolites contribute to the effects, caffeine, chlorogenic acids, cryptochlorogenic acid, and neochlorogenic acid, as well as caffeic acid and trigonelline, were tested in the same manner. TNF release was inhibited by caffeic acid and all three chlorogenic acid isomers, with cryptochlorogenic acid exhibiting the strongest effect. Moreover, cryptochlorogenic acid also distinctly decreased the release of IL-6 and MCP-1, which was not affected by any other pure substance examined. Therefore, the conformation of 4-caffeoylquinic acid appears to be advantageous for influencing the inflammatory mediator release compared to its structural isomers 3- and 5-caffeoylquinic acid. 

Besides caffeine, chlorogenic acids are considered as main bioactive substances in coffee [20], with a number of health-promoting effects, like anti-diabetic and anti-hypertensive activity, but also regulatory action in inflammatory conditions [21,22,23,24]. Structure–activity dependencies of the pharmacological effects of the isomers have scarcely been examined, so far. Anti-inflammatory properties have also been described for their metabolite caffeic acid [24].

Previously suggested anti-inflammatory and immune modulatory activity for the well-investigated stimulant caffeine [25,26,27] and the phytoestrogen and antidiabetic compound trigonelline [28,29,30] could not be confirmed by our investigations, as the two alkaloids did not influence inflammatory mediator release in the applied model.

In conclusion, our findings underline that, beyond its adsorptive effects, coffee charcoal exhibits distinct pharmacological properties, which derive from a spectrum of secondary plant metabolites and support the therapeutic use in inflammatory diseases, especially when applied in a herbal combination in lower dosages. Chlorogenic acids—in particular, cryptochlorogenic acid—appear as pivotal bioactive compounds and can be taken into consideration as active marker substances in the future. Further research, including conclusive definition of the remaining plant substances and the utilization of additional pharmacological models, e.g., regarding other cell types and in vivo settings, is needed to fully unravel the anti-inflammatory mechanisms of coffee charcoal. The observed distinction of the effects of chlorogenic acid isomers provides an interesting starting point for in-depth investigation of the structure–activity relationship.

## 4. Materials and Methods

### 4.1. Substances

Reference substances (caffeine, ferulic acid, caffeic acid, trigonelline hydrochlorid, chlorogenic acid, cryptochlorogenic acid, p-coumaric acid, theobromine, theophyillin, cafestol; purity 99%, HPLC-grade), phorbol-12-myristate-13-acetate (PMA), budesonide (BUD), lipopolysaccharides from *Escherichia coli* O111:B4 (LPS), thiazolyl blue (MTT), and *N*,*N*-Dimethylformamide (DMF) were obtained from Sigma-Aldrich (Taufkirchen, Germany). Neochlorogenic acid (purity ≥99%) was supplied by Extrasynthese SAS (Genay Cedex, France). The cell culture supplies RPMI-1640, fetal bovine serum (FBS), penicillin–streptomycin (P/S), and Dulbecco’s phosphate buffered saline (DPBS) were provided by Biowest (Nuaillé, France). Sodium dodecyl sulfate (SDS) was obtained from Thermo Scientific (Schwerte, Germany) and activated charcoal (*Carbo medicinalis*) from Hevert-Arzneimittel GmbH & Co. KG (Nussbaum, Germany).

### 4.2. Plant Extraction

A coffee charcoal extract was prepared by infusion of 20 g powdered coffee charcoal from roasted beans of *Coffea arabica* L. (*Carbo Coffea* EB 6, batch-no. JB0142) provided by Lomapharm^®^ (Emmerthal, Germany) with 150 mL boiling purified water for 15 min. Subsequently, the extract was lyophilized and stored light-protected at –20 °C until further use for phytochemical and pharmacological characterization.

### 4.3. HPLC and LC/MS Characterization of the Extract

An HPLC analysis of the extract (5 mg/mL; 25 µl injection volume) was conducted with a Dionex Ultimate 3000 HPLC System (Thermo Scientific) with a 717plus Autosampler and 996 photodiode array detector (PDA; Waters, Eschborn, Germany) using reference substances. Gradient elution with methanol 0.1% (eluent A, VWR, Darmstadt, Germany) and aqueous formic acid 0.1% (eluent B, VWR, Darmstady Germany) was performed as follows: 0–5 min 5% eluent A (isocratic); 5–60 min 5% eluent A→100% eluent A; 60–75 min 100% eluent A (isocratic); 75–85 min 100% eluent A→5% eluent A; flow rate: 1 mL/min, 40 °C. 

For the HPLC–MS analysis, an Agilent 1100 system (Santa Clara, CA, USA) consisting of binary pump, autosampler, and variable wavelength detector with a Nucleodur 100-5-C18 ec column (250 mm 4 mm; Macherey-Nagel, Düren, Germany) operated at 25 °C was used for separation. Spectra were recorded with a coupled Bruker Esquire 3000plus (ESI–MS) system (Billerica, MA, USA) in negative and positive ion mode.

### 4.4. Cell Culture

The human leukemic cell line THP-1 [31] (ATCC, TIB-202) was used to model macrophage function. The cells were cultured in RPMI-1640 supplemented with 10% FBS and 1% P/S at 37 °C, 5% CO_2_ humidified air.

For the cytokine release experiments, the cells were seeded in 24-well cell culture plates (Falcon; 0.5 million cells per well) and differentiated into macrophage-like cells using 100 ng/mL PMA for 48 h. After the differentiation period, the cells were treated with various concentrations of the test substances (coffee charcoal extract: 1–500 µg/mL; pure substances: 0.01–100 µM). Budesonide (1 nM) served as a positive control for anti-inflammatory activity. Concomitantly, 100 ng/mL LPS was applied as inflammatory stimulus.

### 4.5. Cytokine Quantification

The cytokine content in the cell-free supernatant was quantified using respective BD OptEIA™ Human TNF, MCP-1 and IL-6 ELISA Sets (Becton Dickinson, Heidelberg, Germany) according to manufacturer’s instructions after 4 h (TNF) and 24 h (IL-6, MCP-1).

### 4.6. Cell Viability Testing

A subsequent MTT assay was performed after 24 h treatment to evaluate cell viability throughout the experiments. After removal of the supernatant, the cells were incubated with 500 µL MTT solution (0.3 mg/mL in DPBS) at 37 °C for 2 h. Resulting formazan salt was dissolved with 500 µL lysis buffer (20 g SDS, 40 mL DMF, ad 100 mL aqua dest, pH = 4,7) overnight. The formazan content was quantified spectrophotometrically at 570 nm with an Infinite^®^ 200 PRO plate reader (Tecan, Crailsheim, Germany).

### 4.7. Scanning Electron Microscopy

Scanning electron microscopic (SEM) images were obtained with the dual beam microscope (DBM) Nova NanoLab 200/600 (FEI Company, Graefelfing, Germany) in the low voltage mode at 5 kV. Activated charcoal and coffee charcoal particles were fixated on a double-sided adhesive tape and sputter-coated with gold to avoid sample charging.

### 4.8. Data Analysis

Spectrophotometric data from cytokine release experiments and cytotoxicity testing were processed with Microsoft Excel 2013. Statistical analysis was performed with GraphPad Prism 6 using an ordinary one-way ANOVA, followed by Dunett’s multiple comparisons test. Observed protein release and metabolic activity were normalized with the LPS-stimulated, untreated control set as 100%. Maximum inhibition was calculated from the normalized values and concentration–response curves based on non-linear regression yielded the half-maximal inhibitory concentrations (IC50) for each treatment, with statistically significant distinction (*p* < 0.05) towards LPS-control. Based on the maximum inhibition, the plant substances were categorized into four categories: No effect (−), <40% mild inhibitory effect (+), <70% medium inhibitory effect (++), ≥70% strong inhibitory effect (+++), and color-coded accordingly from white to dark green for better visualization.

## Figures and Tables

**Table 1:** Summary of the chromatographic, UV-Vis spectroscopic and mass spectrometric characteristics of the plant compounds identified in coffee charcoal extract.

Peak	RT [min]	*λ*max [nm]	*m*/*z*	Ion	Compound
1	4.1	**200**, 263	138.12	[M + H]^+^	Trigonelline
2	32.778	244, **325**	**352.96** 706.84	[M − H]^−^ [2M − H]^−^	Neochlorogenic acid (3-caffeoylquinic acid)
3	39.743	244, **325**	190.92 **352.98** 707.13	[quinic acid-H]^−^ [M − H]^−^ [2M − H]^−^	Chlorogenic acid (5-caffeoylquinic acid)
4	40.093	218, **272**	138.12 163.08 **195.08**	[M + H–OCNCH_3_]^+^ [M + H–CH_3_OH]^+^ [M + H]^+^	Caffeine
5	40.646	244, **325**	190.95 **352.96** 707.13	[quinic acid-H]^−^ [M − H]^−^ [2M − H]^−^	Cryptochlorogenic acid (4-caffeoylquinic acid)
6	44.807	238	172.95 192.96 **366.99** 734.89	[quinic acid–H–H_2_O]^−^ [ferulic acid–H]^−^ [M − H]^−^ [2M − H]^−^	Feruloylquinic acid
7	46.266	235	172.93 190.92 **366.98** 735.15	[quinic acid–H–H_2_O]^−^ [quinic acid–H]^−^ [M − H]^−^ [2M − H]^−^	Feruloylquinic acid
			**334.97** 703.03	[M − H]^−^ [M + feruloylquinic acid–H]^-^	Caffeoylquinolactone

Peak numbers according to the chromatogram shown in Figure 1; RT = retention time, main peak in bold.

**Table 2:** Summary of inhibitory effects of caffeine, caffeic acid, chlorogenic acid isomers, and trigonelline (0.1–100 µM) on LPS-induced inflammatory mediator release from THP-1 macrophages.

Mediator	TNF		IL-6		MCP-1	
Treatment	MI [%]	IC50 [95% CI] [µM]	MI [%]	IC50 [95% CI] [µM]	MI [%]	IC50 [95% CI] [µM]
Caffeine	-	-	-	-	-	-
Caffeic acid	70	24 [14.45–38.83]	-	-	-	-
Chlorogenic acid	69	31 [18.58–44.44]	-	-	-	-
Cryptochlorogenic acid	92	20 [13.01–26.35]	68	21 [5.741–30.62]	73	12 [3.707–21.30]
Neochlorogenic acid	69	28 [17.97–39.43]	-	-	-	-
Trigonelline	-	-	-	-	-	-

MI = maximum inhibition, IC50 = half maximal inhibitory concentration. effect size: - + ++ +++.
